# The relationship between guarding, pain, and emotion

**DOI:** 10.1097/PR9.0000000000000770

**Published:** 2019-07-22

**Authors:** Temitayo Olugbade, Nadia Bianchi-Berthouze, Amanda C de C. Williams

**Affiliations:** a University College London Interaction Centre (UCLIC), University College London, London, United Kingdom; b Research Department of Clinical, Educational & Health Psychology, University College London, London, United Kingdom

**Keywords:** Pain behavior, Chronic pain, Anxiety, Kinesiophobia

## Abstract

**Introduction:**

Pain-related behavior in people with chronic pain is often overlooked in a focus on increasing the amount of activity, yet it may limit activity and maintain pain and disability. Targeting it in treatment requires better understanding of the role of beliefs, emotion, and pain in pain behavior.

**Objectives:**

This study aimed to clarify the interrelationships between guarding, pain, anxiety, and confidence in movement in people with chronic pain in everyday movements.

**Methods:**

Physiotherapists rated extent of guarding on videos of people with chronic pain and healthy controls making specific movements. Bayesian modelling was used to determine how guarding was related to self-reported pain intensity, anxiety, and emotional distress, and observer-rated confidence in movement.

**Results:**

The absence of guarding was associated with low levels of pain, anxiety, distress, and higher movement self-efficacy, but guarding behavior occurred at high and low levels of each of those variables. Guarding was not directly dependent on pain but on anxiety; the relationship between pain and guarding was mediated by anxiety, with a high probability. Nor was guarding directly related to the broader distress score, but to self-efficacy for movement, again with a high probability.

**Conclusion:**

Pain-related guarding is more likely to be effectively addressed by intervention to reduce anxiety rather than pain (such as analgesia); more attention to *how* people move with chronic pain, rather than only *how much* they move, is likely to help to extend activity.

## Abstract 2

Supplemental Digital Content is Available in the Text.

## 1. Introduction

The original description of pain behavior by Fordyce^21^ consisted of guarded movement, vocal signals (such as verbal reports of pain, requests for help, or paraverbal cues such as moaning), and functional limitations (such as absence from work). Each behavior was described with different contingencies of positive and negative consequences within the operant reinforcement system understood to maintain pain behaviors. This understanding was incorporated into the fear-avoidance model,^10,47^ in which cognitive appraisal (mainly catastrophizing) is required for the development of fear that in turn generates avoidance. An alternative formulation, although compatible with the cognitive and behavioral models, was that of Sullivan et al.^41^ (see also Ref. 21), who described pain behaviors as communicative (facial and verbal/paraverbal expression) or protective against risk of future injury or pain, or delayed recovery. The so-called protective behaviors can increase pain and restrict movement,^41^ and include limping and guarding, which are clearly visible (ie, communicate) to observers and may attract adverse characterological judgements.^2,24^ Protective behaviors also involve particular patterns of muscle overactivity, during or after action,^12,22,46,48^ associated with self-rated fear of movement.^42,43^

Investigations of relationships of specific pain behaviors with pain intensity and fear of movement are rare. Guarding, defined as “behavior that is aimed at preventing or alleviating pain” and which includes stiffness, hesitation, and bracing,^33^ has been shown to predict work loss over 3 months^33^ in injured workers. It is associated with self-reported pain intensity,^16,44^ but may itself contribute to the persistence of pain.^22,33^ Our previous studies^3^ showed higher interrater reliability for guarding than for other protective behaviors for videos of people with chronic pain doing a range of movements. This study is part of a longer-term project to build automated systems that can detect pain behaviors and so emulate a good physiotherapist in providing informational and emotional support to encourage self-management of chronic pain in everyday life. The aim of this study was to identify the relationships between guarding, pain, anxiety, and confidence in movement in people with chronic pain that restricted their activities.

## 2. Methods

An existing data set, EmoPain (http://www.emo-pain.ac.uk/),^3,27^ was used to investigate the relationship between the estimates of guarding and self-efficacy, and ratings of general emotional distress, anxiety, and pain of people with chronic pain while engaged in physical activities. The study had ethical approval (UCLH 12/LO/1520, 12/0078, & UCLIC/1516/012/).

### 2.1. Materials and methods

#### 2.1.1. Participants

Participants were either people with chronic low back pain (“patients”) or healthy volunteers (“controls”). Potential participants with low back pain were identified by health care staff at a large pain management centre of a London hospital or recruited through social media. Those who agreed had a brief psychological interview in which the MINI psychiatric interview^37^ was used to exclude major psychiatric comorbidity and severe depression. Inclusion criteria required low back pain for more than 6 months; exclusion criteria were use of mobility aids, past joint replacement, arthrodesis or limb amputation, neuropathic pain, spinal stenosis, cardiovascular or respiratory disease, learning disability, poor understanding of English, or pregnancy. Fifteen female and 7 male patients with a mean age of 51 years provided informed consent for videotaping their movement, but 3 did not provide consent to share their videos, and an additional 2 suffered technical failures; so, 17 patient videos were used for coding. Fourteen male and 14 female controls were recruited among research staff and their friends; they had no history of chronic low back pain, and had a mean age of 37 years. Of these, 6 videos were of insufficient quality and one suffered technical failure, leaving 21 controls.

For exercise recording, participants wore a motion capture suit (Animazoo IGS-190) and EMG (BTS FreeEMG 300), both wireless systems, with an array of video cameras, one of them capturing a wide-angle view, and so full-body movements of the participants. This camera had a resolution of 1024 × 1024 pixels and a frame rate of 58 fps, and the area was lit from multiple directions (further details in Ref. 3). In the study reported here, only the video data were used.

The exercises were a set of basic actions agreed by physiotherapists, with expertise in treating chronic low back pain, to place manageable demands on the lower back. Each had 2 levels of difficulty and a minimum of 2 trials, one at each level of difficulty, was recorded. Three exercises from a set of 7 were used in this study: sit-to-stand, forward trunk flexion with arms horizontal and extended forward, and full trunk flexion pointing the hands towards the toes. Forward flexion was either performed without (low challenge) or holding a 2-kg dumbbell in each hand (high challenge). Similarly, 3 successions of sit-to-stand were performed either self-paced (low challenge) or at a prompt (high challenge). After each exercise, patients reported pain intensity (0 = no pain and 10 = extreme pain) and anxiety (0 = no anxiety and 10 = extreme anxiety). Overall emotional distress was self-rated before doing the exercises, using the Hospital Anxiety and Depression Scale (HADS).^9,50^

#### 2.1.2. Physiotherapist raters

All videos of patient or control exercise were segmented into clips, each participant performing a single instance of an exercise. We recruited UK physiotherapists with experience working with chronic pain patients to label these clips with respect to guarding behavior and self-efficacy levels.^18,27^ To have each video clip annotated by 4 physiotherapist observers, and to minimize time demands on each, 30 raters were required. The experience of the recruited physiotherapists since qualification ranged from 1 to 36 years (median 12 years) and their pain management experience from less than 1 to 32 years (median 5 years).

### 2.2. Scales and measurement

#### 2.2.1. Ratings by physiotherapists

Each video clip consisted of a patient or control performing one instance of the 3 exercises at high or low challenge, with video clips of a mean of 14 subjects (patient or control) per physiotherapist participant (a video set). Each video clip lasted approximately 1 minute. Video clips for controls were included for contrast but their data were not used in the analysis. Video sets were randomly assigned to add up to about an hour of labelling per physiotherapist, with each video set rated by 4 physiotherapists. The proportion of clips were: sit-to-stand (73% of clips), forward trunk flexion (19% of clips), and full trunk flexion (8% of clips). The video clips were shown on a laptop computer with a (diagonal) screen size of 15.5 inches, although there was some variation in actual size of video frame images because different recording devices were used to make the videos and so different players were used in labelling. All were shown mute to minimise distraction from auditory cues.

Previous annotation of the data set for guarding produced low levels of interrater agreement^3^ despite joint training. The problem emerged from the high resolution of the labelling, such that labelling was (and so agreement calculated) frame by frame, and while raters agreed on occurrence of behaviors, they rarely marked the same start or end in the identical event. Such fine-grained annotation was unnecessary and so was revised in this study.

For each clip, raters noted on an Excel worksheet, identified only by ID number without identifying patient or control, the following:

(1) Guarding, presence/absence: guarding was defined as “stiff, interrupted, or rigid movement while moving from one position to another”^16^ (p. 366); stiffness and rigidity were highly correlated in previous ratings;
(2) pain, none/low/high, but raters found this task very difficult and it was not used in the analysis;
(3) movement self-efficacy, described on the rating sheet as confidence, rated low/medium/high.

Physiotherapists also rated overall:

(1) confidence in estimating guarding, pain, and self-efficacy, from 0 (not at all confident) to 6 (completely confident), rated before (median 5, range 3–6) and after (median 4, range 1–5.5) rating videos;
(2) difficulty in estimating guarding, pain, and self-efficacy, from 0 (not at all difficult) to 6 (extremely difficult), after rating only (median 2, range 1–5).

#### 2.2.2. Patient data

As described, the database also contained patients' ratings of pain intensity (0–10) and of anxiety about the exercise (0–10), for each exercise at each challenge level. Overall distress score was obtained from the HADS after dropping one item from the total, providing a possible total score from 0 to 39. The excluded item, *I can sit at ease and feel relaxed*, is likely to elicit pain-related rather than affect-related responses in people with chronic pain.^29,32^ Because the HADS was only completed once by each patient, it was replicated for each exercise instance for analysis.

### 2.3. Analysis

A combination of standard statistical analysis techniques and Bayesian modelling was used to investigate the relationships between the 5 variables: self-reported pain intensity, anxiety level, and emotional distress, and observer-rated guarding and self-efficacy level.

#### 2.3.1. Agreement between raters

Agreement between raters for both guarding and movement self-efficacy was calculated using one-way random, absolute agreement, average-measures intraclass correlation.^25^ Intraclass correlation for guarding was 0.72 for sit-to-stand, 0.63 for forward trunk flexion, and 0.71 for full trunk flexion, all in the good range.^7^ As reported in earlier work,^27^ intraclass correlation for movement self-efficacy was 0.81 for sit-to-stand and full trunk flexion, and 0.70 for forward trunk flexion. Disagreement was mostly between estimates of *medium* and *high* levels of movement self-efficacy.

#### 2.3.2. Relationships between guarding, pain, distress, and movement self-efficacy

Data from healthy participants was excluded, and patient data used for each exercise instance for which there was at least one rating of pain intensity, anxiety, or HADS distress (in addition to the guarding and movement self-efficacy observer ratings). This provided 99 instances from the 17 patients (mean 6, minimum 1, maximum 9). Guarding ratings were coded per rater as 1 (present) or 0 (absent) and the sum across the 4 raters for each exercise instance, from 0 to 4, was used as the guarding score for that exercise. The mean score was 3, SD 1. The score for movement self-efficacy was computed by taking the median of estimates across the 4 raters, where low = 1, medium = 2, high = 3: the mean was 2, SD 1. Mean pain intensity was 5/10 (SD 3, range 10); mean pain anxiety was 1/10 (see Supplementary Table 1 for further description of the spread of anxiety levels, available at http://links.lww.com/PR9/A47), with a range of 9, and mean HADS total was 18 (SD 7, range 28).

Using IBM SPSS Statistics 22, linear relationships between each of pain intensity, anxiety level, distress, movement self-efficacy, and guarding were explored using Spearman rank order correlation. Then, the distribution of each of the first 4 variables in relation to guarding scores was inspected. Finally, a Bayesian network was used to develop an integrated model incorporating the pairwise relationships between all 5 measures. Bayesian network modelling was preferable to multiple regression-based techniques because of the lack of independence of the instances in the data set. A Bayesian model has a graph structure with nodes that represent variables and line connections that describe relationships between them, without circular relationships. This graph structure is built from data using the joint probability distribution/density of the variables, satisfying the Markov condition.^26^ We used the hill-climbing algorithm (Russell and Norvig^35^), which is a greedy score-based search algorithm, to build the Bayesian model for our data based on experimentation. It outperformed the Grow-Shrink Markov Blanket^23^ and the Incremental Association Markov blanket^45^ algorithms in predicting guarding (mean squared error = 1.4, with 5-fold cross-validation). We used the Bayesian Information Criterion (BIC),^36^ which is the log likelihood of any model and also includes a term that penalizes for graph structure complexity, as the scoring function for model selection.

## 3. Results

### 3.1. Relationships between guarding and pain, anxiety, distress, and movement self-efficacy

Results are reported of the Spearman correlation with visual exploration, and of the Bayesian modelling.

#### 3.1.1. Correlational models

Correlations between variables are shown in Table 1. All 4 cognitive and affective measures (pain intensity, anxiety level, self-efficacy, and overall emotional distress) were found to be significantly correlated (all *P* < 0.0001) with guarding, and with one another. The highest correlation was ρ = −0.86 between guarding and self-efficacy, likely influenced by both items being ratings by observers.

**Table 1:**
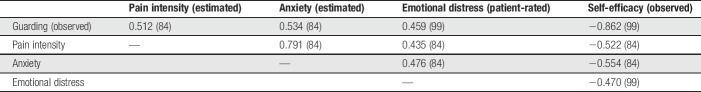
Pairwise Spearman correlation coefficients (number of observations) for guarding, cognitive, and affective scores; all *P* < 0.0001.

**Figure 1.**
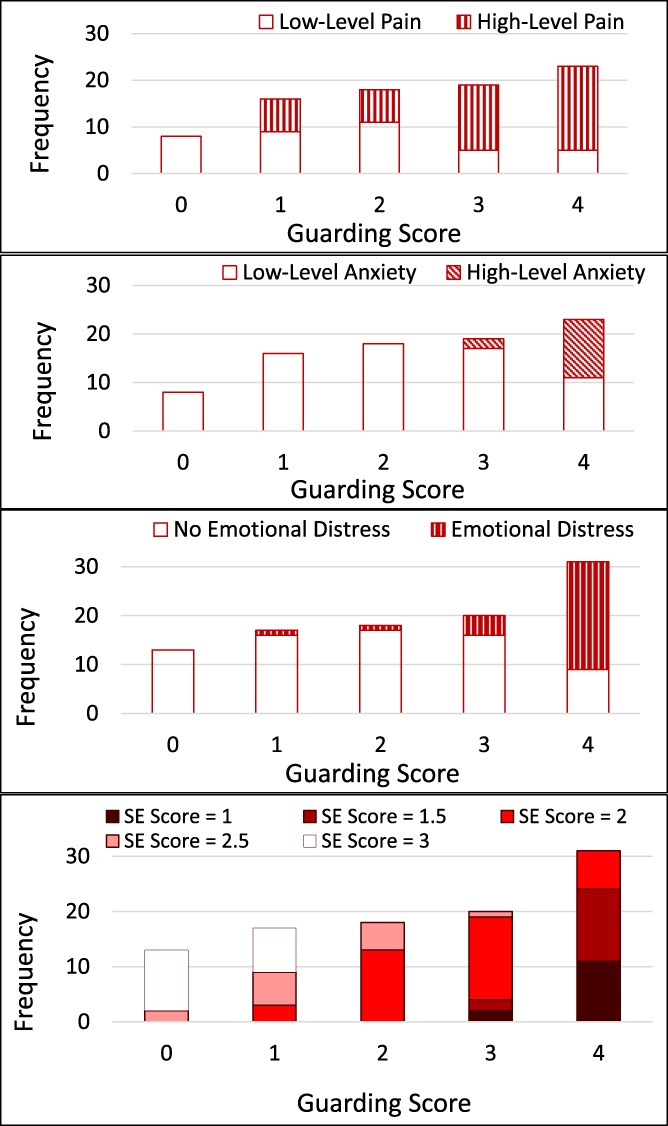
Frequency distribution of guarding scores in people with chronic pain for high and low self-reported pain, anxiety, and emotional distress, and observer-rated movement self-efficacy (SE).

When pain and anxiety were dichotomised as lower (<5/10) and higher (≥5/10), and the HADS distress total as lower (≤19/39) and higher (>19/39), exercise instances where no guarding was observed were predominantly lower pain, lower anxiety, lower distress, and better-than-medium level movement self-efficacy, as shown in Figure 1. Instead, video clips where exercises were judged as definitely showing guarding, scored 4, included both lower and higher levels of pain, anxiety, HADS distress scores, and movement self-efficacy ratings.

#### 3.1.2. Bayesian model

**Figure 2.**
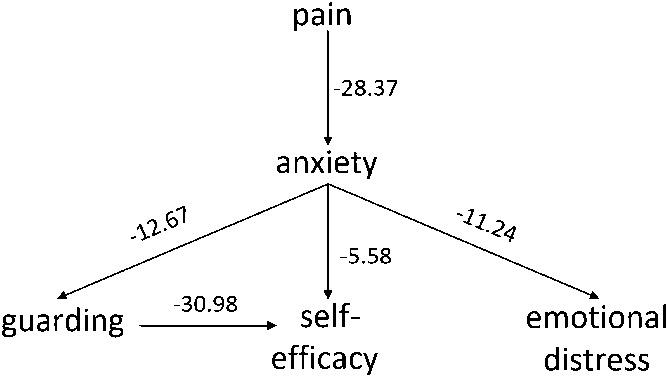
Bayesian network showing independencies between guarding, pain intensity, anxiety, overall distress, and observed movement self-efficacy.

The Bayesian model developed is shown in Figure 2. Bayesian networks are not reliable as causal models,^19^ but indicate conditional independence (between variables with indirect arrow connections, eg, emotional distress and guarding, or pain intensity and guarding, in Fig. 2). The number on each edge of the graph represents the decrease in the BIC (ie, score) of the graph if the edge were removed from the graph. Although the units are arbitrary, guidelines suggest that a difference greater than 10 is highly meaningful,^15^ and so they may be interpreted as the “strength” of the relationship between variables. Thus, guarding is not directly dependent on pain intensity, but its relationship with pain intensity is mediated by anxiety level. Nor is guarding directly related to overall distress. Although this relationship might be influenced by different modes of measurement, it indicates a different relationship between guarding and overall distress (here, a mixture of anxiety and anhedonia) to that between guarding on movement and anxiety about that specific movement. Last, estimated movement self-efficacy is related to patient-rated anxiety about the movement, supporting the meaningfulness of the estimate. Movement self-efficacy is also related to guarding; although the model structure learning algorithm constrained the emergent link to a single (and more likely) direction, from guarding to self-efficacy, it is possible that a minor link exists in the other direction. In fact, this may be the case for all the relationships found.

The strongest relationship found was from guarding to movement self-efficacy, and the next strongest from pain intensity to anxiety level. The strengths of these relationships may be because the 2 constructs, in each pair, were rated by the same person (observer for the former and patient for the latter). However, it does also suggest a high probability of anxiety in the presence of pain, and a higher probability of lower self-efficacy with guarding behavior.

## 4. Discussion

The main finding from this study is that anxiety, and not pain, directly predicted guarding. Pain only predicted guarding indirectly, mediated by anxiety. This serves as an important confirmation of parts of the fear and avoidance model,^46^ and recapitulates some of the findings of Thomas and France.^42^ It is also consistent with findings of only weak associations between pain intensity and pain behaviour.^17,20,21^ Although we did not assess catastrophizing, we believe that our findings support the criticisms of Pincus et al.^31^ and of Crombez et al.^10^ that it is not necessary to postulate that catastrophic thinking mediates between pain and anxiety. Catastrophizing constitutes a process of cognitive appraisal for pain to lead to fear in the fear-avoidance model of chronic pain,^47^ but this is an explicitly human model that is hard to apply to other animals that develop chronic pain and show pain-related behavior such as guarding.^6,40^ Furthermore, the lack of a relationship between guarding and the broader distress score suggests a different behavioral signature for depressed mood in pain than for anxiety.

The findings raise several questions about the construct of pain behavior.^34^ The term “pain behavior” itself may be misleading, implying that the behavior arises from pain, rather than that it is interpreted as indicating pain.^21^ In addition, different pain behaviors may have different emotional and cognitive associations, perhaps consistent with their different functions.^28^ This goes beyond division into protective and communicative behaviors,^41^ which in any case are less distinct than implied, because any visible behavior can be communicative, and help in response to communicative behavior may protect. The term “pain behavior,” or “pain-anxiety behavior,” is likely to be heterogeneous. Function may be far more useful than topology in investigating these pain-related behaviors. Furthermore, even guarding may be heterogeneous in its correlates, as suggested by the plots: there may be more than one cognitive-emotional basis for guarding; so, behavior needs to be studied in the context of patient beliefs, intentions, and emotions. A far larger data set from people with chronic pain is required to investigate this. Similar criticisms pertain to lack of confidence, or self-efficacy for movement, which in this study was strongly associated with more guarding. Self-efficacy is usually studied as a global construct of confidence in doing a range of activities despite pain,^1,11,14^ despite some concerns about its possible heterogeneity.^31^

These findings, with others on a similarly detailed level examining movement and anxiety,^22,42^ suggest that rehabilitation may need to focus on *how* patients move, and not just *how much*.^13^. Current pain management practice of personalised education about the patient's pain condition, goal-setting, and graded increase towards those activity goals^49^ will not necessarily correct subtle or gross muscular habits that not only constrain movement but may contribute to deconditioning and vulnerability to injury.^42^

This project formed part of a larger initiative to develop helpful wearable technology^38,39^ for people with chronic pain, to monitor, prompt, encourage, and record activity, by learning associations and key features of the individual's behavior and physical and emotional challenges, and providing timely, personalised information and feedback in real-life activities and settings (www.emo-pain.ac.uk). This enabled people with chronic pain to apply strategies during functional activity^38^; physiotherapists additionally identified fear of movement as an important target.^39^ Such interventions are valued both by clinicians^30^ and by patients,^4^ providing insights into and support for everyday functioning. Clinicians identified it as an opportunity for more realistic assessment of the effects of therapy, and a way for patients to monitor their progress; patients reported feeling more in control, facilitating pain self-management.

Without intervention, people with chronic pain tend to increase rest, reduce activity, and use analgesics to try to control their pain,^5^ and in our sample, we witnessed many counterproductive behavioral habits that demanded considerable effort and risked increasing pain, even in simple activities such as standing from a seated position (eg, moving the feet as far forward as possible at the start of this movement^27,28^). It is hard to disrupt such habits by occasional physiotherapy sessions, and most systems built for physical rehabilitation do not address the emotional barriers to and influences on movement that are so important in chronic pain,^11,31,47^ nor the habits of movement that are intended to protect. Over time, these habits are associated with greater limitation in movement, and less confidence, while reversing them is associated with reduced disability and better function.^17^

The study had several limitations. Among our variables, pain and anxiety were assessed at each event, whereas distress was a broad overall scale. Associations between ratings were likely to be stronger when made by the same rater, patient, or physiotherapist. The rating of guarding by physiotherapists may have incorporated other behaviors of concern to them, increasing noise in the observational data. Distress is a broad term and we need to understand better which aspects of distress affect guarding and which do not, adding more detailed self-report (of self-statements and emotional state) and physiological variables. We also need to ascertain the beliefs that underlie protective anxiety that generates guarded rather than free movement because those beliefs are a potential target for therapeutic endeavour. Last, the number of participants and observations was lower than ideal, given the intrinsic variability in movement and in cognitive and emotional influences; this may have led to chance associations being given undue weight in the model.

Despite these concerns, we believe that this study contributes to better operationalisation and taxonomy of pain-related behaviors than those currently available^8,41^ and their cognitive and emotional correlates. Finer-grained studies of behaviors and the associated beliefs and emotional states in people with chronic pain,^17^ and longitudinal studies of behavior in relation to disability, will enable us to build smart and effective companion technology that addresses psychological and social well-being alongside physical rehabilitation.

## Disclosures

The authors have no conflict of interest to declare.

This work was funded by the Engineering and Physical Sciences Research Council (EPSRC) grant “*Pain rehabilitation: E/Motion-based automated* coaching,” EP/H017178/1. T. Olugbade was funded by the 2012 Nigerian Presidential Special Scholarship Scheme for Innovation and Development.

The work was part of the PhD of the first author: http://discovery.ucl.ac.uk/10045652/.

## Supplementary Material

SUPPLEMENTARY MATERIAL

## Appendix A. Supplemental digital content

Supplemental digital content associated with this article can be found online at http://links.lww.com/PR9/A47.
