# Supplementary material for: The relationship between guarding, pain, and emotion
Source: Pain Rep. 2019 Jul 22;4(4):e770. doi: 10.1097/PR9.0000000000000770 (PMC6728010; doi:10.1097/PR9.0000000000000770)
Supplement: SUPPLEMENTARY MATERIAL [file painreports-4-e770-s001.docx]

Supplementary Table 1. Frequency distribution of anxiety intensity in people with chronic pain

| Anxiety Level | Frequency of self-report | Number of Patients |
| --- | --- | --- |
| 0 | 40 | 11 |
| 1 | 13 | 2 |
| 2 | 14 | 3 |
| 3 | 1 | 1 |
| 4 | 2 | 2 |
| 5 | 5 | 3 |
| 6 | 1 | 1 |
| 7 | 0 | 0 |
| 8 | 0 | 0 |
| 9 | 8 | 1 |
| 10 | 0 | 0 |
